# Rhizosphere Signaling: Insights into Plant–Rhizomicrobiome Interactions for Sustainable Agronomy

**DOI:** 10.3390/microorganisms10050899

**Published:** 2022-04-25

**Authors:** Fatima Jamil, Hamid Mukhtar, Mireille Fouillaud, Laurent Dufossé

**Affiliations:** 1 Institute of Industrial Biotechnology, Government College University, Lahore 54000, Pakistan; fatimajamil000@gmail.com; 2 CHEMBIOPRO Chimie et Biotechnologie des Produits Naturels, Faculté des Sciences et Technologies, Université de la Réunion, F-97490 Sainte-Clotilde, Ile de La Réunion, France; 3 CHEMBIOPRO Chimie et Biotechnologie des Produits Naturels, ESIROI Département Agroalimentaire, Université de la Réunion, F-97490 Sainte-Clotilde, Ile de La Réunion, France; laurent.dufosse@univ-reunion.fr

**Keywords:** rhizosphere, microbial metabolites, plant–microbe signaling, quorum sensing, phytohormones, defense priming, sustainable agriculture

## Abstract

Rhizospheric plant–microbe interactions have dynamic importance in sustainable agriculture systems that have a reduced reliance on agrochemicals. Rhizosphere signaling focuses on the interactions between plants and the surrounding symbiotic microorganisms that facilitate the development of rhizobiome diversity, which is beneficial for plant productivity. Plant–microbe communication comprises intricate systems that modulate local and systemic defense mechanisms to mitigate environmental stresses. This review deciphers insights into how the exudation of plant secondary metabolites can shape the functions and diversity of the root microbiome. It also elaborates on how rhizosphere interactions influence plant growth, regulate plant immunity against phytopathogens, and prime the plant for protection against biotic and abiotic stresses, along with some recent well-reported examples. A holistic understanding of these interactions can help in the development of tailored microbial inoculants for enhanced plant growth and targeted disease suppression.

## 1. Introduction

The rhizosphere is a most captivating environment, which harbors a variety of microorganisms that are deeply involved in plant–microbe communication. This high-density niche allows plants to interact with associated microorganisms through chemical signals that are produced in response to specific stimuli, which in turn activate many regulatory mechanisms [1]. Rhizospheric regions possess higher bacterial activity than non-rhizospheric regions. The composition of a microbiome is determined by different biotic and abiotic factors, i.e., the climate, type of soil, and chemical signals that are produced by the plant and its associated microbes [2,3].

Plants and microbes have diverse interactions that involve close interactions, either positive or negative, including mutualism (symbiosis), parasitism, and commensalism [4]. Positive interactions include those with microorganisms, e.g., rhizobia, plant growth-promoting rhizobacteria (PGPR), and mycorrhiza, which result in beneficial outcomes, such as growth promotion, nutrient accessibility, and protection against abiotic and biotic environmental stresses [5,6,7]. On the other hand, plant interactions with microbial pathogens result in negative outcomes, i.e., plant diseases [8,9]. The interaction between both partners depends on specialized signaling molecules or chemical signals that are also significant in cooperative, as well as competitive, microbial behavior [10,11]. The chemical cues or secondary metabolites act as mediators in plant–microbe and microbe–microbe communication and also trigger plant responses [12].

In the past decade, progress has been made in the understanding of the types of chemical signals that are responsible for controlling the activities of plants and associated microbes [13,14,15]. The most studied signaling compounds are N-Acyl homoserine lactones (N-AHLs), which are produced by a variety of bacterial taxa and regulate quorum sensing and pathogenicity within a bacterial population [16,17,18]. Likewise, plant roots secrete a variety of metabolites as exudates, including photosynthetically derived carbon compounds, e.g., organic acids, vitamins, flavonoids, polysaccharides, amino acids, and sugars. These root exudates create an enriched environment for the rhizomicrobiome to interact and increase diversity based on the composition of the exudates [15,19,20]. Moreover, plants release secondary metabolites against pathogens and insects that act as defensive signals [21,22]. Plants use adaptive strategies to enhance the defensive capacity of their innate resistance to biotic and abiotic factors by interacting with beneficial microbes [23,24]. To unravel the process of microbial interaction with plants, an understanding of the types of chemical communication between all members is necessary. Thus, the known microbial community and their interactions could help in the optimal use of beneficial microbes for better plant growth.

Some of the literature on different signaling molecules that participate in the development of interactions within the rhizosphere for enhanced plant growth has been reviewed [15,25,26,27,28]. However, much still needs to be explored in terms of the significance of these interactions as far as microbial or chemical diversity and the understanding of signaling molecules are concerned. This review aims to enlighten our understanding of rhizosphere signaling in plant–microbe communication, both cooperative and competitive, and its significance in plant productivity and the development of sustainable agriculture systems. Nonetheless, the signaling compounds have a prodigious potential to escalate plant functions when they are understood in depth.

## 2. Rhizosphere: A Pool of Plant–Microbe Signaling

**Figure 1:**
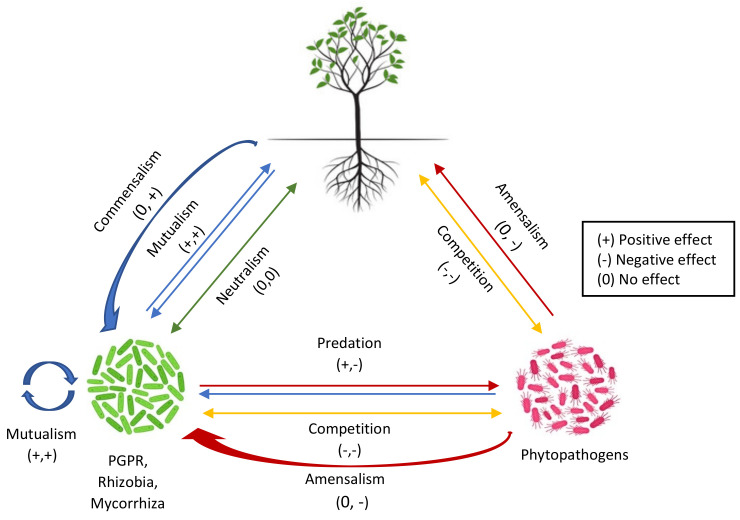
Schematic representation of interactions between rhizo-microorganisms and plants.

The rhizosphere serves as a hotspot for diverse microbial activity. It is an intricate ecosystem comprising nutrient-rich soil that surrounds the plant roots, which provides a pool for plant–microbe communication. The term “Rhizomicrobiome” is defined as a microbial community that is present in the rhizosphere [29]. A variety of microorganisms reside within the rhizosphere, including bacteria, fungi, nematodes, protists, and invertebrates [11]. Most of the microbiome studies within the context of rhizosphere signaling have been focused on the bacteria and fungi that make up the major portion of the rhizosphere microbiome. However, viruses that infect bacteria, which are known as phages, have an influence on the dynamics of the rhizosphere microbiome. The most abundant known phages in the soil virome include *Microviridae, Siphoviridae*, and *Podoviridae* [30]. The diversity and amount of identified viruses in the rhizosphere is much less, i.e., 2700 less than bacterial abundance, which amounts to around 10^10^ species per g of soil [31,32]. The complete biological entity comprising a plant and its associated microbial community, i.e., the symbionts (facultative and obligate) and microbes that have parasitic relationships with the host plant, is referred to as a “holobiont” [33]. The cascade of intricate chemical signals develops a communication within the rhizosphere that facilitates various mechanisms in a holobiont, i.e., root–root interactions, biofilm formation, nutrient acquisition, microbiota development, and resistance against pathogens [34,35,36]. Different types of interactions between microbes and plants are depicted in Figure 1.

According to the “rhizosphere effect”, this region is associated with a lesser microbial diversity but a higher bacterial abundance compared to bulk soil [37]. The recruitment of microbes within the rhizosphere directly depends on the soil properties, soil type, and plant metabolites [38]. Different plant genotypes and physicochemical soil properties develop specific environments for the selection of a promising microbiome [10,34]. Plant-derived metabolites or root exudates play a significant role in the shaping of the rhizomicrobiome by modifying the soil chemistry around the plant roots and serving as substrates for the growth of specific microbiota [39]. The root exudates differ both in quality and quantity depending on the type, nutritional status, and growth stage of the plant [40]. These root exudates, including both primary and secondary metabolites, contain 10–16% of the total plant nitrogen and 11% of the photosynthetically fixed carbon that actively tune the microbiota in the microbial reservoir that is within the vicinity of the plant roots [41]. Another factor that affects the rhizomicrobiome is the cellular response that is shown either by the microbes or the plants, which results in transformation, catabolism, and resistance to the chemical that is being sensed [11]. The release of root exudates into the rhizosphere by plants selects the desired microbial community by attracting it while deterring harmful communities, which in turn allows plants to be adaptable. This selection process for microbiota is known as “niche colonization” [42,43].

Signaling in the rhizosphere can be categorized into two major types based on the direction of the communication, i.e., the inter- and intraspecies microbial signaling and the interkingdom signaling between microbes and plants.

### 2.1. Inter- or Intraspecies Signaling among Microorganisms

Microbes in the rhizomicrobiome interact with each other by producing signaling molecules that adjust their gene expression [11]. Inter- or intraspecies communication among microbes occur via the quorum sensing mechanism, which involves cell density-dependent coordination. Quorum sensing (QS) is the cell-to-cell signaling process that takes place through the synthesis, release, and detection of chemical signals [44,45]. These chemical signals are also known as autoinducers (AIs) and they regulate the gene expression of some bacterial functions upon the recognition of the signal by the recipient, i.e., biofilm formation, adhesion, motility [46,47,48], propagation, virulence, metabolism [49], and symbiotic association [1].

Quorum sensing signals in bacteria are categorized into two groups: acyl-homoserine lactone (AHLs or AI-1), which is found in Gram-negative bacteria, and autoinducer peptides (AIPs), which are found in Gram-positive bacteria; and autoinducer type 2 (AI-2), which has the traits of both AHLs and AIPs and is found in both Gram-negative and Gram-positive bacteria [50,51]. N-acyl homoserine lactone (N-AHL) has been majorly reported in various Gram-negative bacteria, including *Burkholderia* sp., *Pseudomonas syringae*, *Pseudomonas putida*, *Pseudomonas chlororaphis*, *Erwinia* sp., and *Serratia* sp. (Table 1) [52,53]. As well as AHLs, a diverse range of signals have been reported in Gram-negative bacteria, i.e., fatty acid methyl esters, 2-alkyl-quinolones, furanone, and γ-butyrolactones [16,50,54].

Another type of QS bacterial signals is antibiotics, which have been reported for intra- or interspecies communication [67]. A recent study revealed some antibiotic signals in a bacterial strain that resided in the tobacco rhizosphere, *Lysobacter capsica*. The strain possessed biocontrol potential due to its production of antifungal and antibiotic signals, i.e., cyclic lipodepsipeptides and cyclic and polycyclic tetramate macrolactams [68]. Fungi also produce quorum sensing signals that communicate with bacterial species, e.g., γ-heptalactone, tyrosol, γ-butyrolactone, dodecanol, and farnesol [69]. Fungal and bacterial interaction through QS signals creates a competition for existence among the associated microbes in the rhizosphere in terms of infecting the host [70].

Another important class of signaling molecules is volatile organic compounds (VOCs). Microbial VOCs are synthesized and released for long-distance communication among a microbial community and also in microbe–plant interaction [71]. VOCs are low molecular weight lipophilic compounds (100–500 Da) that are produced by various bacterial and fungal species through distinct metabolic pathways that are specific to the species genotype [72]. The VOCs of bacterial origin include alkanes, ketones, alkene, terpenoids, and sulfurs [73,74]. VOCs play an important role in microbe–microbe communication by serving as antimicrobial QS signaling molecules and influencing microbial activity, i.e., virulence, stress resistance, and biofilm formation [75,76]. These attributes are exemplified as a low concentration of nitric oxide (NO) influences the biofilm formation in bacterial species such as *P. aeruginosa*, *B. licheniformis*, and *S. marcescens* [77]. Apart from this, VOC signals have been reported to regulate plant growth (root architecture and hormonal signaling) and plant immunity against biotic and abiotic stresses [71]. Additional studies will further unravel other signaling mechanisms that are associated with microbial VOCs.

Hence, these intricate signaling mechanisms among rhizospheric microorganisms have a key role in the shaping of the rhizomicrobiome by recruiting specific microbes through inter- or intraspecies communication.

### 2.2. Interkingdom Signaling

Microbes and plants interact with each other via interkingdom signaling, which can influence plant growth by either inducing or suppressing gene expression. Interkingdom signaling can be subdivided into two categories based on the stimulus direction: microbe–plant signaling and plant–microbe signaling.

#### 2.2.1. Microbe–Plant Signaling

In microbe–plant signaling, microbes produce and emit signals that induce symbiotic interactions with the plant. Microbial signals that are of rhizosphere origin can trigger definite changes in the plant transcriptome. As in plants, phytohormones are also produced by PGPR. These plant growth-stimulating signals can regulate the developmental processes of plants and can also provide plants with resistance to abiotic and biotic stresses. Many PGPRs, including *Azospirillum* spp., *Bacillus amyloliquefaciens*, *B. muralis*, *B. thuringiensis*, *Rhizobium* spp., and Pseudomonas spp., have been reported to produce a diverse group of phytohormones, e.g., auxin, abscisic acid, salicylic acid, cytokinin, gibberellin, and strigolactones [78,79,80,81]. A recent study in Nature reported three newly isolated strains of *Phoma* spp. in the rhizosphere of *Pinus tabulaeformis*. These strains secreted stress the resistance substance abscisic acid (ABA) when under drought stress, which triggered drought resistance mechanisms in the pine tree and also stimulated its antioxidant activities [82].

Most of the literature on microbe–plant communication has been focused on beneficial interactions, such as the induction of plant growth and plant defenses against biotic and abiotic stresses. Rhizospheric microbes that have plant-friendly associations include mycorrhiza, rhizobia, plant growth-promoting bacteria, and fungi (PGPR or PGPF) [11]. Microbial signals are recognized by plants as microbe-associated molecular patterns (MAMPs), i.e., flagellin, chitin, and lipopolysaccharides, via pattern recognition receptors (PRRs) that trigger a local defense through a hormonal signaling network, which in turn produces immune responses [83,84,85].

**Figure 2:**
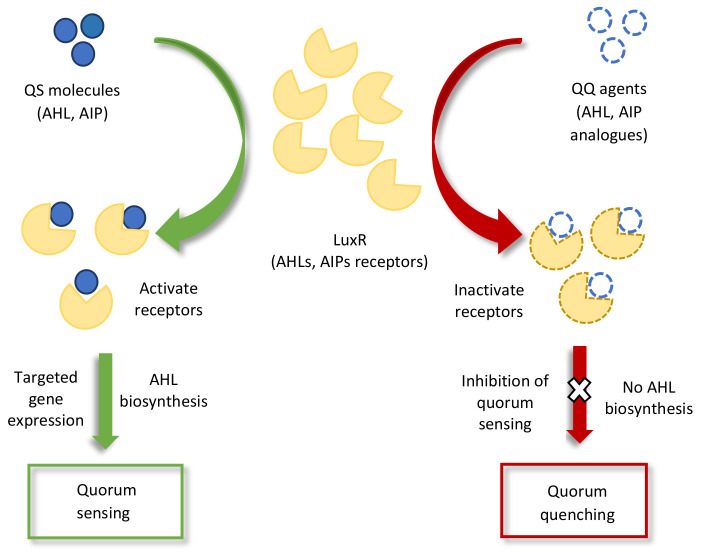
Schematic representation of quorum sensing (QS) and quorum quenching (QQ) inhibition of signal perception pathways. The binding of QQ agents to the LuxR receptors either inactivates quorum sensing receptors or reduces the quantity of receptors in the QS molecules for targeted gene expression.

Quorum sensing signals have also been reported in interkingdom communication. Bacterial QS signals that are perceived by plants elicit various plant responses, e.g., AHLs (N-butanoyl homoserine lactone and N-hexanoyl homoserine lactone), which aid in the development of symbiotic association with plants and enhance root growth by modifying the hormonal levels in the plant [46,86]. Another bacterial QS molecule, DSF, stimulates innate immunity (the detection of harmful microbes via PRRs) in different plants [87]. Some other examples of QS sensing in interkingdom communication is presented in Table 1. As well as causing physiological changes in plants, bacterial QS molecules also provoke the plants into secreting molecules that mimic the QS molecules of pathogenic microbes [88,89]. For example, the p-coumaric acid that is produced by garlic [90], the isothiocyanate sulforaphane that is produced by broccoli [91], the curcumin that is produced by turmeric [92], and the patulin that is produced by fruits (apple, banana, pear, grape, etc.) [93]. Studies on AHL mimicry have shown that the mimicry molecule of bacterial AHL (AHL analog) specifically interferes with QS regulatory pathways and can either stimulate or inhibit the gene expression of the original QS receptor or host, as illustrated in Figure 2 [94,95]. This type of interference in microbial QS signaling for attenuating pathogenicity is known as quorum quenching (QQ) [44,96,97]. A recent study identified a quorum quenching mechanism in a novel bacterial strain, Acinetobacter sp. strain XN-10, which degraded the QS molecules of the AHL family that was acting against the QS-mediated pathogenicity. The strain suppressed the pathogenicity of *Pectobacterium carotovorum* subsp. *carotovorum* (Pcc), thereby protecting tissue maceration in potatoes, cabbage, and carrots [98]. Another study reported a quorum quenching defense mechanism against *Pseudomonas aeruginosa* infection in plants. Upon the interaction of the plant with *P. aeruginosa*, the plant-secreted QQ molecule (rosmarinic acid) mimicked and competed with the QS pathogenic signal (C4-HSL) and stimulated QS-mediated responses that provide disease protection to the plant, i.e., virulence factors and biofilm formation [88].

Rhizospheric bacteria have been reported to produce antimicrobial signaling compounds. These compounds stimulate systemic resistance in plants against phytopathogens by altering hormonal pathways, e.g., the di-acetyl phloroglucinol (DAPG) that is produced by *Pseudomonas* sp. [99]. It is common that microbially produced VOCs can induce plant growth and resistance to biotic stress [72,100]. Plants perceive and respond to various VOC signals of PGPR or PGPF origins, such as undecanone and heptanol. For example, 2-heptanol and 2-undecanone, which are produced by *B. subtilis* and *B. amyloliquefaciens*, promote the growth of *Arabidopsis thaliana* when cultivated with these PGPR strains [101,102].

#### 2.2.2. Plant–Microbe Signaling

Plants serve as residences for microbial communities, which include mutualists, commensals, and parasitic microorganisms. Plants respond to the microbial signals by secreting chemical compounds, which are known as root exudates. These include low molecular weight compounds (organic acids, sugar, aliphatic acids, fatty acids, amino acids, flavonoids, and secondary metabolites) and high molecular weight compounds (proteins and mucilage) [20,34,103]. The plant–microbe interaction through the secretion of phytochemicals affects the biology of the rhizosphere [104]. The secreted chemicals attract rhizospheric microbes toward the plant roots and develop either pathogenic or symbiotic interactions with them. The most studied plant–microbe interaction is the symbiotic relationship of legumes with nitrogen-fixing bacteria. The cascade of signals that is produced upon plant–microbe interaction forms root nodules. Rhizospheric microbes feed on these nodules and in turn provide the plant with an available form of nitrogen. Plant signals for nodule formation have been extensively studied over the last decade [15,105].

A recent work demonstrated pathogenic plant–microbe interaction through a broccoli plant induced defense system, which released root exudates upon interaction with fungal species. The root exudates (isothiocyanates (ITC) and glucosinolates (GLS)) showed antifungal potential against the rhizospheric microbes *Pseudomonas syringae, Sphingomonas suberifaciens*, and *Fusarium oxysporum* [106]. The chemical complexity and specificity of the root exudates depend on the genotype of the plant. The chemical signals of root exudates attract specific microorganisms for interaction [34,35]. For example, cucumber secretes citric acid, which attracts *Bacillus amyloliquefaciens*, whereas bananas secrete fumaric acid, which attracts *B. subtilis* N11, but both interactions result in biofilm formation [107]. Therefore, specific root exudates recruit specific microbial communities within the rhizosphere. Phytochemicals are also capable of stimulating the QS signaling mechanism in microbes, e.g., the flavonoids that are produced by legumes upregulate the gene expression of AHL genes in rhizobia [108].

Plants also release volatile organic compounds (VOCs) from different parts: leaves, flowers, fruits, and roots [109]. These include terpenoids, fatty acids, phenylpropanoids, and amino acids that constitute approx. 1% of the total plant secondary metabolites. VOCs can easily cross plant membranes and be released into the atmosphere or soil. In soil, they attract root colonizing pathogens and inhibit their growth [110].

**Figure 3:**
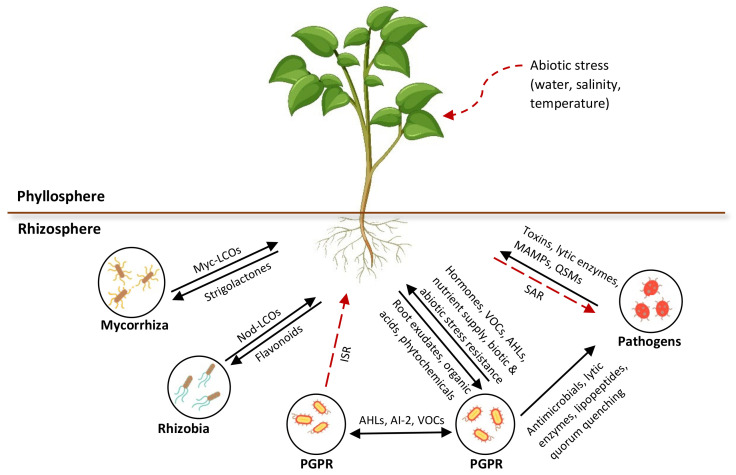
Overview of rhizosphere communication in intra- or interspecies signaling among microorganisms and interkingdom signaling between microbes and plants. Myc, mycorrhizal; LCOs, lipo-chitooligosaccharides; Nod, nodulation; VOCs, volatile organic compounds; Ais, autoinducers; AHLs, N-acyl homoserine lactone; QSMs, quorum sensing molecules.

Plant secretions, including root exudates, volatiles, and strigolactones, are widespread signaling compounds that bind with bacterial receptor proteins and elicit a response in microbes that regulates gene expression. The signaling molecules that have been identified so far are known to influence the developmental and defense processes of plants. These phytochemical compounds are responsible for shaping or adapting the rhizomicrobiome due to their specificity and complexity. However, many of these chemical compounds and their target functions are yet to be explored. The rhizosphere signaling mechanisms that are involved in the interactions between microorganisms and between the microorganisms and plants are represented in Figure 3.

## 3. Significance of Signaling in Plant–Rhizomicrobiome Interaction

**Figure 4:**
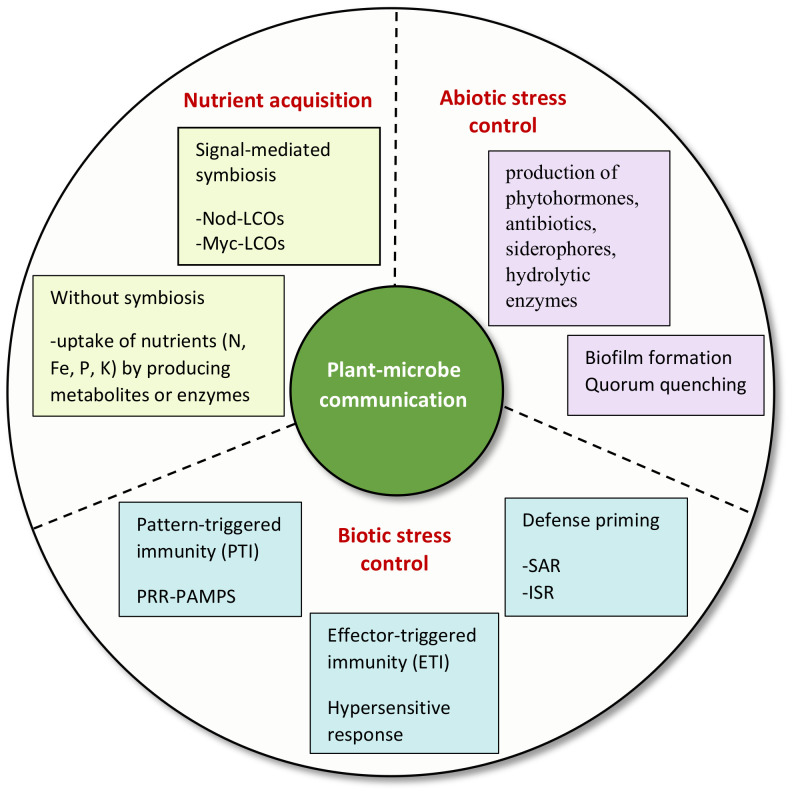
Summary of significant factors of rhizosphere signaling. SAR, systemic acquired resistance; ISR, induced systemic resistance; LCOs, lipo-chitooligosaccharides; Nod, nodulation; Myc, mycorrhizal; PRR, pattern recognition receptor; PAMPs, pathogen-associated molecular patterns.

The plant–microbe signaling in the rhizosphere contributes toward sustainable agronomy. Some features of rhizosphere signaling that have significant importance are elaborated here. A summary of the significant factors of rhizosphere signaling is shown in Figure 4.

### 3.1. Nutrient Acquisition for Phytostimulation

A plant can acquire nutrients from the rhizomicrobiome in two ways: either through symbiotic or non-symbiotic interactions with rhizospheric microorganisms.

#### 3.1.1. Signal-Mediated Symbiosis

Plants are known to develop synergistic interactions with the associated rhizomicrobiome. Nutrient acquisition is a crucial consequence of these interactions as once the symbionts have established the association, a cascade of signals results in the continuous exchange of nutrients between both symbionts. The most studied interkingdom signal-mediated interactions are the mutualistic associations between rhizobia and legumes and between AMF and non-vascular plants. These interactions have been extensively studied throughout the last decade; however, research is still ongoing with regards to signal-mediated symbiosis.

- Rhizobia–legume symbiotic interactions

Signal-mediated symbiosis plays a key role in nutrient acquisition for plants. Rhizobia provide a reduced form of nitrogen, i.e., ammonia, to plants and in exchange, the plants provide dicarboxylates to the rhizobia [111].

The symbiotic interactions between rhizobia and legumes are initiated after the secretion of phytochemicals by the roots of the host plant, i.e., flavonoid derivatives [112]. Flavonoids have been well studied regarding their role in legume–rhizobia symbiosis. They can accumulate auxin in the root tissues, which facilitates the nodulation process [113]. The flavonoids act as a chemical attractant for rhizobia and guide the rhizobial cells into activating the nodulation genes (nod, noe, and nol), which in turn synthesize nodulation (Nod) factors, i.e., lipo-chitooligosaccharides (LCOs) [114]. LCOs are major signaling molecules for the initiation of nodule formation, which is perceived by receptors that are present in the root epidermis of the host plant (known as kinases). This results in nodule formation in the roots of the legumes [15,115,116]. The legume secretions are rhizobia-specific and attract specific rhizobial species. Likewise, the rhizobia response is specific to the flavonoids, i.e., distinct LCOs diffuse through rhizobia and activate certain nodulation genes that are specific to legume species in order to develop an accurate symbiotic interaction [117,118]. LCOs can promote plant growth by modulating legume root architecture, which in turn enhances nutrient acquisition. This process has been implicated in the increase in the number of root hairs for increased nutrient uptake [119]. This behavior, including an increase in root surface area, length, and hair number, was observed in *Arabidopsis thaliana* that was inoculated with *Bradyrhizobium japonicum* [120]. As with flavonoids, some other signaling molecules have also been reported to activate nod genes, e.g., chalcones, betaines, vanillin [121], jasmonate, and aldonic acid [122]. Thus, these signaling compounds regulate the gene expression of particular partner symbionts for successful symbiosis.

The neighboring microbes of rhizobia in a phytomicrobiome assist in nodule formation by producing synergistic consortia that play an important role in the alleviation of abiotic stress, nutrient uptake, and disease resistance [118]. The co-inoculation of PGPR has shown drastic plant growth-promoting effects, e.g., *Mesorhizobium cicero* that was co-inoculated with *Bacillus* sp. and *Enterobacter aerogenes* demonstrated significantly enhanced root nodulation, uptake of nitrogen and phosphorous, and total protein content in the chickpea plant [123].

- Mycorrhizal symbiotic interactions

Mycorrhizal symbiosis involves the interactions between arbuscular mycorrhizal fungi (AMF) and land plants. The signaling pathways are quite similar to those in rhizobia–legume symbiosis. Plant secretions of strigolactones, which are known as branching factors (BFs), send signals to the AMF that, upon recognition, release mycorrhizal lipo-chitooligosaccharides (Myc factors) that are responsible for the development of the symbiotic interactions between plants and AMF [124,125]. As well as acting as AMF stimuli, strigolactones are plant hormones that are capable of interfering with auxin transport [126]. Intriguingly, AMF penetrate through the epidermal cells of plant roots after perceiving plant signals. There, they start to differentiate into highly branched structures called arbuscles. AMF colonize, either intercellularly or intracellularly, within the cortical tissues that have a key role in the nutrient exchange between both symbionts [127,128]. A complex cascade of signals enhances the nutrient uptake of the roots, such as nitrates, amino acids, immobile ortho-phosphates, starch, and calcium, using specialized transporters. This improves the metabolic pathways of the plant while the host plant fulfills the carbohydrate requirements of the AMF in return [129,130,131].

Recently, studies have reported a tripartite interaction between three symbionts: AFM–rhizobia–plant (ARP). This tripartite interaction increases nitrogen fixation, helps the plant to survive in drought conditions [132,133], and enhances root soluble sugar content and dry weight, as well as increasing the total number of nodules [133,134]. Some of the literature also considered tripartite symbiosis to be an efficient interaction for plant growth [98,135,136,137]. Further research is required to understand the gene expression profiles and metabolic events that are associated with ARP tripartite interactions. Meta-genomics would provide better ways to monitor and understand the role of each partner that is involved in the symbioses within the rhizosphere.

#### 3.1.2. Nutrient Acquisition without Symbiosis

Plants can acquire nutrients without symbiosis with rhizobia or AMF. Various microbes meet nutrient requirements via the production of certain metabolites [19,138,139]. During iron deficiency, certain microbes colonize the roots of a plant in iron deficit soil and produce chelating compounds to sequester iron from the soil. Bacterial colonization induces the gene expression that is responsible for iron uptake in plants in response to iron deficiency [139,140,141]. The phosphate requirements of the arabidopsis plant are provided by endophytic bacteria *Colletotrichum tofieldiae* via the translocation of phosphate through the roots [142]. The rhizospheric and endophytic consortium of *Bacillus* sp. and *Pseudomonas* sp. Has been shown to significantly enhance the phosphate solubilization efficiency of wheat cultivars that were growing in phosphate deficient soil [143]. Similarly, microbes other than rhizobia meet the nitrogen demands of non-leguminous plants [19,144]. The decomposition of organic matter by rhizospheric microbes enhances plant productivity and soil fertility by providing nutrients to plants. For example, lignocellulolytic fungi, such as, *Pleurotus ostreatus, Phanerochaete*, and *Trichoderma harzianum*, and bacteria, including *Pseudomonas* sp., *Sporocytophaga* sp., *Cytophaga* sp., and *Streptomyces* sp., degrade plant biomass and release nutrients into the soil, which are taken up by the plants that are growing in nutrient-poor soil [145,146]. The soil phages or virus-like particles (VLPs) affect the availability of nutrients to plants by modulating mutations in microbial phylotypes or interfering with the structure and diversity of the bacterial population. These effects generate mutations in rhizodeposits and aid in nutrient uptake (N, S, and P) by the plants. Despite the lower abundance of the lysogenic virus community than the bacterial community, viruses play their role in the resilience of the rhizosphere microbiome [30].

### 3.2. Regulation of Plant Immunity against Phytopathogens

Biotic stresses cause a 30% loss in crop yield and a 15% loss in food worldwide due to various pathogen attacks, i.e., bacteria, fungi, viroids, viruses, protists, nematodes, and insects [147]. PGPR induce an innate immune response in plants against the phytopathogens in a sequential manner, i.e.: (i) perceive the stress stimulus; (ii) regulate defense responses by activating defense-related signaling pathways; and (iii) defense priming that prepares the plant to respond against the stress after recognition by the PGPR [148,149]. The perception of the stimulus by the plant triggers a local immune defense response that is translated into a systemic defense response, which is mediated by hormonal signaling pathways [150].

#### 3.2.1. Pattern-Triggered Immunity (PTI)

**Figure 5:**
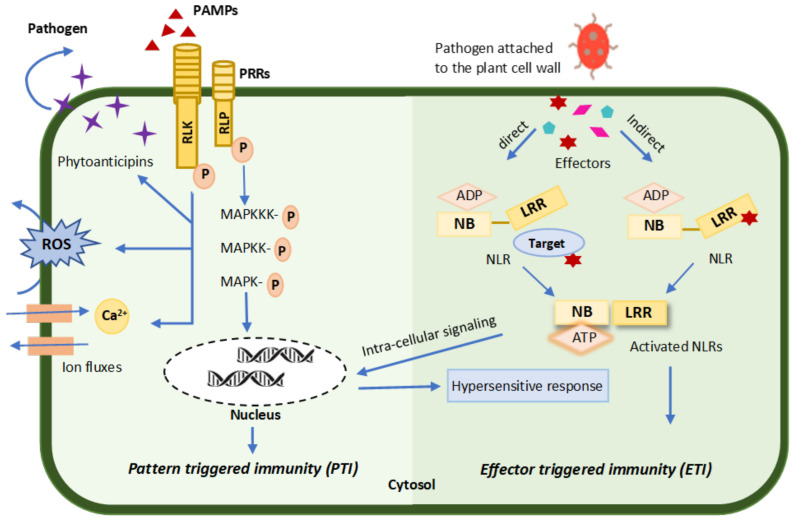
Schematic representation of plant immunity against phytopathogens through pattern-triggered immunity (PTI) and effector-triggered immunity (ETI). PTI is activated through the recognition of pathogen-associated molecular patterns (PAMPs) by the pattern recognition receptors (PRRs) in plant cell walls. After PAMP recognition, the plant triggers a signal cascade that further induces multiple intracellular defense responses and activates defense genes. In response to PTI, pathogens secrete effectors that are recognized by nucleotide-binding (NB) and leucine-rich repeat (LRR) receptors (NLRs) and develop another defense layer called ETI. Activated NLRs with many intracellular signaling events trigger the hypersensitive response (HR).

Plant immunity is initiated by host–pathogen interactions, in which plant receptors and pattern recognition receptors (PRRs) recognize the pathogen- or microbe-associated molecular patterns (PAMPs or MAMPs) that induce the resistance mechanisms. After PAMP recognition, the plant triggers signaling modules, i.e., mitogen-activated protein kinases (MAPKs) and calcium-dependent protein kinases (CDPKs), which further triggers a signal cascade that is responsible for the activation of specific transcription factors that lead to the induction of multiple intracellular defense responses. The induction of the signal cascade is either hormone-dependent (MAPKs and CDPKs) or -independent [12,151,152]. The plant signals that make up the signal cascade include methyl jasmonate (MeJA), azelaic acid (AzA), pipecolic acid (Pip), and salicylic acid (SA) signals that regulate defense genes [153,154]. Some phytohormones, such as auxins, cytokinins, gibberellins, and abscisic acid, also take part in this signal cascade [28]. This type of defense response is known as MAMP- or PAMP-triggered immunity [155,156,157] or more generally as pattern-triggered immunity (PTI) [158], which is depicted in Figure 5.

The induction of PTI stimulates intracellular defense responses, including stomatal closure, the deposition of callose, and the induction of reactive oxygen species (ROS) and antimicrobial metabolites (Figure 5) [158,159]. Several AHLs and diffusible signal factors (DSFs) have been identified as quorum sensing signals in phytopathogenic interactions. In the host–pathogen interaction between *Arabidopsis thaliana* and *Sclerotinia sclerotirum*, small sRNAs were abundantly identified in the signal pathways [160]. Plants produce secondary metabolites upon pathogen interaction, which are known as “phytoanticipins” or “phytoalexins” (Figure 5) [157,161]. The biosynthesis of these inducible compounds is regulated by phytohormone signaling pathways, phosphorylation, and defense-related genes [162]. Capsidiol production in *Nicotiana benthamiana* has been shown to be mediated by an ethylene signaling pathway that provides defense against *Phytophthora infestans* [163].

#### 3.2.2. Effector-Triggered Immunity (ETI)

In response to PTI signaling, pathogens resist the plant defense mechanisms by producing effectors that prevent their detection by the host plant. This response mechanism is known as effector-triggered susceptibility (ETS) [83,155,164]. Upon ETS activation, the plant in turn develops another defense layer, which is known as effector-triggered immunity (ETI). ETI is activated upon the detection of pathogen effectors through a specialized intracellular protein, which is known as NB-LRR (nucleotide-binding and leucine-rich repeat domains) [83,156,165]. NB-LRR is encoded by a plant resistance gene (R gene) that mediates resistance to specific phytopathogens, i.e., viruses, oomycetes, fungi, nematodes, and bacteria. It recognizes the specific pathogen effectors either by direct binding or by changing its protein binding site during pathogen–effector binding [165], as depicted in Figure 5. The virulence specificity of pathogen effectors has been as yet undiscovered because several PRRs can recognize specific pathogens and pathogens have evolved effectors that dislocate the defense signaling of plants [166]. This protective mechanism is called the hypersensitive response (HR) [167]. For example, *Phytophthora infestans* produces the effector protein (PexRD2), which increases its susceptibility to *Nicotiana benthamiana* by suppressing the host’s defense signaling [168]. Effector proteins have also been reported to attenuate hormonal signaling, i.e., salicylic acid and jasmonic acid signals induce *P. infestans* colonization and infection in plants [169].

### 3.3. Defense Priming

Plants use adaptive strategies to enhance the defensive capacity of their innate resistance. Warning signals or stimuli, such as pathogens, abiotic stresses, chemicals, PGPR, and arthropods, trigger their innate resistance and prime the plants against secondary stresses. This type of enhanced defense response state is known as priming. During this phase, plants change at transcriptional, physiological, metabolic, and epigenetic levels [23]. Primed plants show induced and more competent responses against stresses [170,171]. Defense priming can be sustained throughout the life of a plant and can be transferred on to the next generation. Defense priming is mediated by two induced signaling resistance pathways: systemic acquired resistance and induced systemic resistance.

- Systemic acquired resistance (SAR)

PTI and ETI trigger long-distance defense signals that induce resistance against the future localized infection via broad-spectrum phytopathogens, which involves the activation of proteins and pathogenesis-related (PR) genes. This type of long-distance immune priming that is induced by a pathogen is known as systemic acquired resistance (SAR). It is a salicylic acid (SA)-dependent pathway [83,153,172]. Salicylic acid is produced by various rhizobacteria that are responsible for activating PR genes, PTI, and ETI in SAR signaling [173].

**Figure 6:**
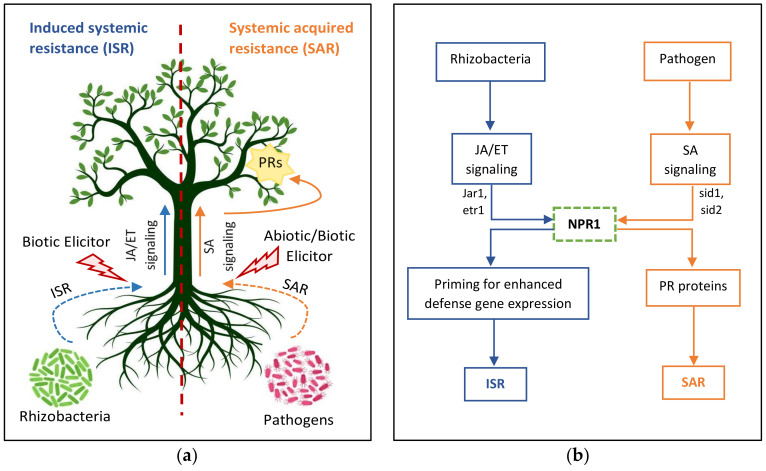
Schematic representation of defense priming: (**a**) a picture depicting induced systemic resistance (ISR), which is induced by plant growth-promoting rhizobacteria that provide resistance to biotic stresses, and systemic acquired resistance (SAR), which is induced by phytopathogens that provide resistance to biotic and abiotic stresses; (**b**) a flowchart showing signal transduction pathway of ISR that are followed by jasmonate (JA) and ethylene (ET) plant hormones, which are encoded by the jar1 and etr1 genes, respectively, whereas SAR is followed by salicylic acid (SA), which is encoded by the sid1 and sid2 genes.

SAR pathways are regulated by the key protein NPR1 (non-repressor of PR genes), which interacts with transcriptional co-factors that in turn induce the expression of pathogen-related proteins that are associated with systemic resistance [174]. SA binds with NPR1 and induces conformational changes in its structure to further expose it to transcriptional factors for PR gene activation (Figure 6) [175]. Jasmonic acid (JA) pathways in SAR protect the plant from necrotrophic pathogens by activating a regulatory protein: COI1 in the form of a ubiquitin complex. The activated protein complex participates in the defense of the plant [174].

SA pathways have been reported to induce the biosynthesis of flavonoids, i.e., pro-anthocyanidins and catechin, which inhibit the proliferation of foliar rust fungus *Melampsora larici-populina* in poplar trees [176]. SA and methyl jasmonate (MeJA) have been shown to mediate the induction of defense-related genes and proteins against the blight disease-causing pathogen *Xanthomonas axonopodis pv. manihotis* in cassava plants [177]. *Trichoderma harzianum* has also been shown to induce systemic resistance via ET, SA, and JA pathways against the cucumber mosaic virus in tomato plants [178]. *Trichoderma viride* has been reported to provide resistance against *Phytophthora infestans* infection via SA signaling in potato plants [179].

- Induced systemic resistance (ISR)

The induction of defense signaling by a beneficial rhizomicrobiome, such as PGPR against phytopathogens and pests, is known as induced systemic resistance (ISR), which is an SA-independent pathway [83,180]. This defense signaling pathway also increases plants’ tolerance of abiotic and biotic stresses and is therefore also known as induced systemic tolerance [181,182]. The core difference between ISR and SAR is the dependency of SAR on the SA synthesis and accumulation by PGPR that activates SA signaling pathways, whereas ISR is SA-independent and includes ET and JA signaling pathways (Figure 6) [14].

Beneficial microbes do not possess virulence genes but they do have the potential to sensitize plants by exploiting them to perceive MAMPs that lead to the activation of ISR [183]. Defense priming by rhizobacteria overlaps under biotic and abiotic stresses.

ISR is regulated by the signaling pathways of phytohormones, including ethylene (ET) and jasmonic acid (JA) [184,185]. The reactive oxygen species (ROS) and reactive nitrogen oxygen species (RNOS) form a complex defense signaling network by stimulating the biosynthesis of JA, ET, and SA [186]. As an example, JA and ET were shown to regulate a defense response by stimulating the lipid transfer protein gene against *Fusarium graminearum* [187]. Another defense response that is induced by JA and ET includes the RSL1 (recombinant *Solanum lycopersicum*) protein that triggers the gene expression of ET and JA against phytopathogens, e.g., *Alternaria alternata* and *Botrytis cinerea* [188]. ISR expression is usually determined by the type of bacterial strain and the plant in which resistance is induced [189]. PGPR consortiums have also been reported to induce ISR. For instance, a PGPR consortium was shown to trigger ISR in the cucumber plant by increasing its antioxidants and root vigor index while also regulating the photosynthetic machinery [190]. Moreover, the co-inoculation of AMF with *Pseudomonas* sp. reportedly enhanced the antioxidant machinery in the leaves and the phosphatase activity in the roots of lettuce plants under drought stress [191].

PGPR products that elicit defense priming include VOCs, LOCs, AHLs, ACC deaminase, antibiotics, siderophores, and lipopolypeptides [192,193]. The literature has reported that *Bacillus amyloliquefaciens* produced lipopolypeptides that primed the lettuce plant against *Rhizoctonia solani* [194]. AHLs from *Acidovorax radicus* N35 reportedly primed the defense response by stimulating the production of flavonoids, i.e., lutonarin and saponarin, in barley [195]. Furthermore, a siderophore-producing *Pseudomonas putida* mutant strain was shown to have an efficient biocontrol ability over the siderophore-deficient *Pseudomonas aeruginosa* mutant strain in controlling *Fusarium* wilt in tomato plants [196].

Many rhizobacteria have been reported to have parasitic traits due to the production of cell wall degrading enzymes, e.g., *Stenotrophomonas maltophilia* secretes proteases that have a parasitic action on *Bursaphelenchus xylophilus* (nematode) and *P. ultimum* (oomycetes) [197,198]. Some phages play a role in controlling the infection of a plant through bacterial phytopathogens. A recent study that was published in Nature reported that a combination of phages lessened the infection of tomato plants that was caused by *Ralstonia solanacearum* [199].

### 3.4. Regulation of Plant Defenses against Abiotic Stresses

Plants experience about 70% damage due to various abiotic stresses, including temperature, drought, nutrient deficiency, salinity, and heavy metal toxicity. A rhizomicrobiome manages to ameliorate abiotic stresses through induced systemic tolerance via microbe–plant signaling, which involves biochemical and physiological changes in the plants [200]. Many PGPR have been reported to ameliorate abiotic stresses by adopting defense-related mechanisms, i.e., biofilm formation or the production of phytohormones, antibiotics, siderophores, hydrolytic enzymes, and quorum quenching compounds [201,202]. Rhizobacteria that possess the genetic capability to mitigate abiotic stresses include *Caulobacter*, *Rhizobia*, *Serratia*, *Flavobacterium*, *Erwinia*, *Chromobacterium*, *Burkholderia*, *Methylobacterium*, *Trichoderma*, *Micrococcus*, and *Pseudomonas* [203,204,205].

Plants manage to respond to abiotic stresses through a complex signal cascade that is activated following the perception of stress stimuli by the receptors or sensors that are in the plant cell walls. The sensed signals are then translated into intracellular signals through secondary messengers, i.e., calcium ions, nitric oxide, sugars, cyclic nucleotides, inositol phosphate, and ROS. These secondary messengers transduce the signaling pathways of the stress signals [206,207]. As a result, various physiological and metabolic responses become activated during different stages of plant development, such as the synthesis of jasmonic acid, ethylene, salicylic acid, abscisic acid [208,209], flavonoids, phenolic compounds [210], antioxidants, and osmolytes, as well as the activation of certain transcription factors for the expression of stress genes [211,212].

PGPR-produced phytohormones, mainly ethylene (ET), jasmonate (JT), salicylic acid (SA), abscisic acid (ABA), cytokinin, auxin, and gibberellins, regulate abiotic stress responses in plants. These hormones interact either synergistically or antagonistically to adjust their biosynthesis according to the stress response [213,214]. ABA biosynthesis genes are triggered upon osmotic stress to enhance its production, which is responsible for maintaining water balance through the closure of stomata [215]. ABA acts synergistically with SA and antagonistically with ET to regulate osmotic stress, stomatal movement, and leaf senescence under drought conditions [216]. The exact mechanisms of bacterially produced phytohormones need to be explored further. However, some of the reported hormonal signaling pathways of rhizo-microorganisms in different host plants are presented in Table 2. PGPR can effectively stimulate antioxidant activity in plants under drought and salinity conditions. As a result, plants increase their antioxidant signaling, i.e., the production of redox enzymes and phenolic-like compounds (glycine, proline, and betaine) [217].

Enzymatic pathways, including phosphatases and protein kinases, i.e., calcium-dependent protein kinases (CDPKs), mitogen-activated protein kinases (MAPKs), and receptor-like kinases, have been reported to regulate dephosphorylation and phosphorylation in the management of abiotic stresses in plants [241]. Plants can deregulate the salt-sensitive gene expression under salinity stress via calcium ion signaling pathways [242]. PGPR-mediated stress resistance is involved in these signaling pathways that modulate the transcription factors in order to enhance the expression of stress-responsive genes [232]. For instance, *Bacillus amyloliquefaciens* has been shown to ameliorate the salt stress response in *Oryza sativa* by upregulating MAPK and CDPK pathways [243]. Another study showed an improved salinity stress resistance in wheat plants following inoculation with bacterial strains, i.e., *Enterobacter cloacae*, *Pseudomonas fluorescens*, *Pseudomonas putida*, and *Serratia ficaria*, which elevated the Na^+^/K^+^ ratio [244].

## 4. Conclusions and Future Perspectives

The interaction intricate chemical signaling between plants and their associated microbiome deciphers the communication dynamics that are underway within the rhizosphere and their outcomes in terms of plant productivity and development. Over the last decade, quorum sensing has been studied in the gene expression of both beneficial and harmful interactions between inter- and intramicrobial species and in the interkingdom signaling between plants and PGPR, i.e., nitrogen-fixing bacteria and rhizobia. Most of the plant metabolites that have been reported to date have been characterized by the *Arabidopsis thaliana* plant model. The recent literature suggests that other plants need to be investigated for the analysis of more plant metabolites and chemical cues. Expanding the research on rhizosphere communities will aid in the discovery of new chemical cues and their potential to enhance plant productivity in terms of symbiosis, resistance against environmental stresses, and immunity or defense mechanisms toward phytopathogens. Based on experimental evidence, there is no doubt that plants create an environment that recruits a specific rhizomicrobiome and shape microbial associations that are beneficial for their growth. Therefore, the escalating pressure of the demand for high crop production has opened up new avenues for research on rhizosphere signaling to reduce dependency on synthetic processes and agrochemicals. The alterations in the metabolic pathways of plants, microbes, rhizodeposits, and signaling molecules could be effective in the development of a rhizomicrobiome that is beneficial for effective plant productivity and resistance toward biotic and abiotic stresses, which could ultimately lead to more sustainable agriculture. Moreover, future research can be expanded by using novel “multi-omics techniques”, which encompass genomics, proteomics, transcriptomics, phenomics, metagenomics, metabolomics, metatranscriptomics, metaproteomics, and metagenomics and could reveal multi-layered information about new chemical cues, cellular mechanisms, signaling mechanisms, and plant genes. Furthermore, future research on rhizosphere engineering is essential to dissect the targeted outcomes of the beneficial plant–microbe communications that could broaden the application of sustainable agriculture.

## Figures and Tables

**Table 1:** Characterization of quorum sensing signaling molecules that are produced by different rhizomicrobes with respect to the type of communication within the rhizosphere.

Type of Microbe	Rhizo-Microorganisms	Quorum Sensing Molecules	Type of Communication	Reference
Gram-positive bacteria	*Bacillus subtilis*	ComX pheromone	Inter- and intraspecies	[6]
	*Streptomyces* spp.	Gamma-butyrolactones (A-factor) and methylenomycin furans (MMF1)	Interspecies	[55]
	*Staphylococcus aureus*	Circular oligopeptide	Interspecies	[16]
	*Stenotrophomonas chelatiphaga*	DSF (diffuse signal factor)	Interkingdom (poplar plant)	[56]
	*Bacillus velezensis*	AI-2 synthetase (2-methyl-2,3,3,4-tetrahydroxytetrahydrofuran (THMF))	Interkingdom (maize plant)	[57]
Gram-negative bacteria	*Burkholderia* sp.	N-3-oxo-hexanoyl-homoserine lactone (3OC6-HSL)	Interkingdom (wheat and arabidopsis plant)	[58]
	*Serratia glossinae*	*N*-hexanoyl-L-homoserine lactone (*m*/*z* 200) and *N*-octanoyl-L-homoserine lactone (*m*/*z* 228)	Interkingdom (rice plant)	[59]
	*S* *erratia plymuthica*	*N*-butanoyl-HSL, *N*-hexanoyl-HSL, and *N*-3-oxo-hexanoyl-HSL (OHHL)	Interkingdom (oil seed rape)	[60]
	*Burkholderia graminis* M12 and *B. graminis* M14	*N-(*3-oxododecanoyl)-L-homoserine lactone (3-oxo-C12-HSL or OC12-HSL (where “O” indicates an oxo substitution at the third carbon atom)) and 3-oxo-C14-HSL (OC14-HSL)	Interkingdom (tomato plant)	[61]
	*Serratia marcescens*	N-3-oxo-hexanoyl-homoserine lactone (3OC6-HSL)	Intraspecies and interkingdom (tobacco plant)	[62]
	*Burkholderia* sp. and *Pseudomonas* sp.	*N*-butyryl-homoserine lactone (C4-HSL)	Interkingdom (arabidopsis plant)	[63]
	*Ochrobactrum* sp.	3O-C7-HSL and 3OH-C7-HSL	Interkingdom (bean plant)	[64]
	*Stenotrophomonas maltophilia*	DSF (diffuse signal factor)	Interkingdom (oil seed rape)	[65]
	*Serratia glossinae*	*N*-octanoyl-_L_-homoserine lactone and *N*-hexanoyl-_L_-homoserine lactone	Interkingdom (sesame plant)	[59]
Fungi	*Candida albicans*	Tyrosol, γ-butyrolactone, and farnesol	Intraspecies	[66]

**Table 2:** Amelioration of abiotic stresses by rhizo-microorganisms using different signaling pathways in different plant species.

Stress Type		Host Plant	Rhizo-Microorganisms	Signaling Pathways	Reference
Salinity		*Triticum aestivum*	*Arthrobacter protophormiae* (SA3) and *Dietzia natronolimnaea* (STR1)	IAA, ET	[218]
		*Lycopersicum esculentum*	*Leclercia adecarboxylata*	IAA	[219]
		*Triticum aestivum*	*Dietzia natronolimnaea*	SOS, ABA	[220]
		*Cucumis sativus*	*Burkholdera cepacia*, *Promicromonospora* sp., and *Acinetobacter calcoaceticus*	GA, ABA, SA	[221]
		*Cucumis sativus*	*Trichoderma asperellum*	IAA, ABA, GA	[222]
Drought		*Lolium multiflorum*	*Bacillus* sp. and *Pseudomonas* sp.	ABA	[223]
		*Triticum aestivum*, *Zea mays*	*Bacillus* sp. and *Enterobacter* sp.	IAA, SA	[224]
		*Oryza sativa*	*Pseudomonas fluorescens*	ABA	[225]
		*Nicotiana tabacum*	*Glomus versiforme* (AMF) and *Bacillus methylotrophicus*	ABA, IAA	[226]
Heat		*Eucalyptus grandis*	*Brevibacterium linens*	ET	[227]
		*Lycopersicum esculentum*	*Bacillus cereus*	ET	[228]
		*Arabidopsis*	*Bacillus licheniformis*	JA, ABA	[229]
		*Triticum aestivum*	*Bacillus safensis* and *Ochrobactrum pseudogrignonense*	ROS	[230]
Low temperature		*Triticum aestivum*	*Bacillus velezensis*	ROS, ABA	[231]
		*Oryza sativa*	*Bacillus amyloliquefaciens*	ABA, SA, JA, ET	[232]
		*Solanum lycopersicum*	*Funneliformis mosseae* and *Paraburkholderia graminis*	ROS	[233]
		*Arabidopsis thaliana*	*Burkholderia phytofirmans*	ROS	[234]
Heavy metals	Cd	*Oryza sativa*	*Enterobacter aerogenes*	IAA, ET	[235]
		*Momordica charantia*	*Stenotrophomonas maltophilia* and *Agrobacterium fabrum*	ET	[236]
	Ni, Cd, and Al	*Glycine max*	*Paecilomyces formosus* and *Penicillium funiculosum*	IAA, GA	[237]
	Cd, Cu, Pb, and Ni	*Spartima maritima*	*Bacillus methylotrophicus*, *Bacillus licheniformis*, and *Bacillus aryabhattai*	COX, AOX	[238]
		*Solanum tuberosum*	*Bacillus sp.*	ROS	[239]
	As	*Glycine max*	*Bradyrhizobium japonicum* and *Azospirillum brasilense*	IAA	[240]

IAA, indole acetic acid; ET, ethylene; GA, gibbberellin; JA, jasmonic acid; ROS, reactive oxygen species; ABA, abscisic acid; SOS, salt overly sensitive; COX, cytochrome oxidase; AOX, alternative oxidase.

## Data Availability

Not applicable.

## Abbreviations

PGPR	Plant growth-promoting rhizobacteria
N-AHL	N-Acyl homoserine lactone
AIs	Autoinducers
QS	Quorum sensing
QQ	Quorum quenching
VOC	Volatile organic compound
MAMP	Microbe-associated molecular pattern
PRR	Pattern recognition receptor
DSF	Diffusible factor
ITC	Isothiocyanate
GLS	Glucosinolates
AMF	Arbuscular mycorrhizal fungus
Nod	Nodulation
LCOs	Lipo-chitooligosaccharides
BF	Branching factor
PAMP	Pathogen-associated molecular pattern
MAPKs	Mitogen-activated protein kinases
CDPKs	Calcium-dependent protein kinases
PTI	Pattern-triggered immunity
ROS	Reactive oxygen species
ETS	Effector-triggered susceptibility
ETI	Effector-triggered immunity
NB-LRRs	Nucleotide binding and leucine-rich repeat domains
HR	Hypersensitive response
SA	Salicylic acid
NPR1	Non-expressor of pathogenesis-related genes
SAR	Systemic acquired resistance
JA	Jasmonic acid
ET	Ethylene
MeJA	Methyl jasmonate
AzA	Azelaic acid
Pip	Pipecolic acid
ISR	Induced systemic resistance
ACC	1-Aminocyclopropane-1-carboxylic acid
ABA	Abscisic acid
AOX	Alternative oxidases
COX	Cytochrome oxidase
GA	Gibberellin
SOS	Salt overly sensitive
